# Septic embolic encephalitis after *Staphylococcus aureus* endocarditis of a prosthetic valve in a 57-year-old woman: a case report

**DOI:** 10.4076/1757-1626-2-6653

**Published:** 2009-07-31

**Authors:** Lars Tönges, Sara Pilgram-Pastor, Miriam Puls, Holger Schmidt

**Affiliations:** 1 Department of Neurology, Georg-August-University GöttingenFaculty of Medicine, Waldweg 33, 37073 GöttingenGermany; 2 Department of Neuroradiology, Georg-August-University GöttingenFaculty of Medicine, Robert-Koch-Str. 40, 37075 GöttingenGermany; 3 Department of Cardiology and Pulmonary Medicine, Georg-August-University GöttingenFaculty of Medicine, Robert-Koch-Str. 40, 37075 GöttingenGermany; 4 Department of Medical Psychology and Medical Sociology, Georg-August-University GöttingenFaculty of Medicine, Waldweg 37A, 37073 GöttingenGermany

## Abstract

For prosthetic heart valves the risk of infection is much higher than for native heart valves. During the course of infective endocarditis 20-40% of all patients suffer from cerebrovascular complications such as ischaemic stroke or intracerebral haemorrhage. We present the case of a 57-year-old woman who had undergone surgery to mechanically replace an aortic heart valve 11 months ago and suffered from repeated ischaemic strokes with secondary haemorrhage. The initial antibiotic regimen was ineffective in treating the later diagnosed *Staphylococcus aureus* infection of the prosthetic valve. Escalation of the antibiotic treatment was not able to halt the clinical course that finally led to the patient’s death. The case report emphasizes the importance of early identification of the aetiology of infection in patients with mechanical heart valve replacement. Without rapid and adequate treatment there is a considerable risk for the development of severe neurological sequelae and cardiac failure that can ultimately result in a fatal course of this clinical picture.

## Introduction

Infective endocarditis (IE) is a microbial infection of the endocardial surface of the heart that most commonly consists of an infective vegetation of a heart valve. In Western Europe, the incidence of community-acquired infective endocarditis is 2.2 to 6.2 cases per 100,000 person-years [1]. Regarding only prosthetic valves the risk of infection is much higher accumulating to about 1 percent at 12 months and 3 percent at 60 months after surgery [2,3]. During the active course of IE 20-40% of all patients suffer from cerebrovascular complications such as transient ischaemic attacks, ischaemic strokes or in some cases of intracerebral haemorrhages that are often caused by ruptured cerebral mycotic aneurysms [4,5].

## Case presentation

**Figure 1.**
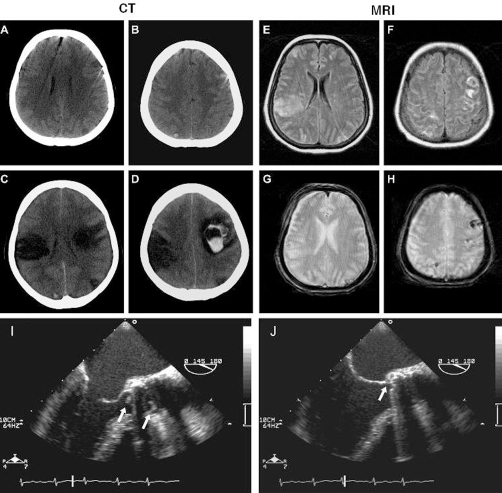
Axial cNECT and cMR images on admission **(A+B, E-H)** and 4 days after onset of neurological symptoms **(C+D)**. **(A+B)** NECT images show initially small cortical bleedings left frontal and right occipital and swollen cortex sections right occipital. **(C+D)** 4 days later the haemorrhage is enlarged, and multiple zones of infarction are visible. **(E+F)** Axial FLAIR weighted images demonstrate bilaterally multiple cortical and subcortical signal hyperintensities representing multiple ischemic lesions. **(G+H)** Axial T2*GRE MR images show microbleedings and haemorrhages within infarcted lesions. Transoesophageal echocardiogram examination shortly after admission **(I+J)**. **(I)** Demonstration of two large oscillating vegetations (arrows), one of 4.5 × 2 mm on the upstream side and one of 4.3 × 7.4 mm on the downstream side of the bileaflet tilting disk valve. **(J)** Closer examination of the mechanical aortic valve shows relevant thickening of the aortic root indicating an evolving ring abscess (arrow).

After acute development of a left sided palsy a 57-year-old Caucasian German woman was referred to us with a preceding 4-day history of high-grade fever, coughing and general weakness. Due to mechanical replacement of the aortic valve 11 months ago, ampicillin and sulbactam had been selected for antibiotic treatment. Initial chest X-ray, transthoracic echocardiography, abdominal ultrasound, and cultures of blood and urine had all been negative for signs of infection. The neurological exam revealed left facial weakness, slurriness of speech, left-sided hemiparesis and hemihypaesthesia whereas clinical examination was normal apart from minor respiratory distress. Blood tests showed a normal white cell count, a low platelet count (60 × 109/l), elevations of creatine kinase (466 U/l), serum troponin T (0.04 μg/l), and an elevated CRP (471 mg/l) while coagulation tests demonstrated an INR of 2.4 (under coumarine treatment) that was normalized rapidly thereafter. The initial brain CT and MRI revealed two secondarily haemorrhaged infarcted areas (Figure 1). The analysis of the cerebrospinal fluid displayed a cell count of 127/μl, a total protein of 1.36 g/l and lactate concentration of 3.5 mmol/l. Because of the severe septic clinical course antibiotic therapy was changed to ceftriaxone, gentamicin and linezolid. Complementary transoesophageal echocardiography showed two major vegetations on the mechanical aortic valve and the development of an aortic ring abscess (Figure 1, I+J). These findings pointed conclusively to a septic embolic encephalitis due to IE. A severe deterioration of the patient’s clinical condition caused by additional intracranial bleedings four days later (Figure 1, C+D) prevented a surgical replacement of the aortic valve. All blood cultures revealed a *Staphylococcus aureus* bacteriaemia resistant to the formerly applied ampicillin but sensitive to gentamicine and linezolid. Although medical therapy was intensified, the patient finally died from cardiac failure.

## Discussion

Mortality in IE patients is strongly elevated if they suffer from cerebrovascular complications. Prostethic valve IE by themselves have a significantly elevated mortality in comparison to native valve IE [6]. Especially in the case of IE due to *S. aureus,* mortality is higher in prosthetic valve IE than in native valve IE. Effects of anticoagulant therapy can be deleterious [7,8]. In accordance with current guidelines for the treatment of brain abscess linezolid was chosen instead of the glycopeptides vancomycin or teicoplanin because the oxazolidinone linezolid has a much higher penetration into the cerebrospinal fluid and was shown to effectively cure staphylococcal CNS infections in several reports [9,10]. Our case demonstrates the importance of early diagnosis and early initiation of an effective antibiotic therapy in IE. It is of utmost importance that these patients are immediately referred to a specialized centre to be evaluated for valve replacement as soon as possible.

## Abbreviations

cMR: cranial magnetic resonance
cNECT: cranial noncontrast-enhanced computed tomography
CNS: central nervous system
CRP: C-reactive protein
CT: computed tomography
FLAIR: Fluid-Attenuated Inversion Recovery
IE: infective endocarditis
INR: international normalized ratio
MRI: magnetic resonance imaging
T2*GRE: T2* Gradient-Recalled-Echo
U/l: Units per litre
